# Quality and Safety of Fresh Chicken Fillets after High Pressure Processing: Survival of Indigenous *Brochothrix thermosphacta* and Inoculated *Listeria monocytogenes*

**DOI:** 10.3390/microorganisms7110520

**Published:** 2019-11-02

**Authors:** Anthoula A. Argyri, Olga S. Papadopoulou, Patra Sourri, Nikos Chorianopoulos, Chrysoula C. Tassou

**Affiliations:** Institute of Technology of Agricultural Products, Hellenic Agricultural Organization—DEMETER, Sof. Venizelou 1, Lycovrissi, 14123 Attica, Greece; olga_papadopoulou@outlook.com (O.S.P.); sourri_p@hotmail.com (P.S.); nchorian@nagref.gr (N.C.); ctassou@nagref.gr (C.C.T.)

**Keywords:** high-pressure processing, *Listeria monocytogenes*, *Brochothrix thermosphacta*, PFGE, poultry, safety, quality

## Abstract

The effect of high-pressure processing (HPP) on *Listeria* *monocytogenes*, the indigenous microbiota and the shelf-life of chicken fillets was evaluated. Chicken fillets were inoculated with different inocula (2, 4, and 6 log CFU/g) of a 4-strain cocktail of *L. monocytogenes*, vacuum-packed, processed or not with HPP (500 MPa/10 min) and stored at 4 °C and 12 °C. Total viable counts (TVC), *L. monocytogenes*, *Pseudomonas* spp., *Brochothrix thermosphacta*, lactic acid bacteria (LAB), *Enterobacteriaceae* and yeasts/molds were determined along with the pH and sensory analysis. Pulsed-field gel electrophoresis (PFGE) was used to monitor the succession of indigenous *Brochothrix* isolates and inoculated *Listeria* strains. The main spoilage microorganism of HPP-treated samples was *B. thermosphacta* detected after 3 days of storage. HPP decreased the inoculated *Listeria* population. For the low and medium inoculum case it was detected throughout the shelf-life at both temperatures in populations near to the detection limit or after enrichment. In the high inoculum case, the pathogen decreased ≥5-log cycles after HPP, while increased subsequently to 1.6 and 4.5 log CFU/g at 4 °C and 12 °C, respectively, by the end of the shelf-life. PFGE showed that *Brochothrix* isolates exhibited a significant diversity among control samples, whereas this was limited for the HPP-treated samples. The survival and distribution of different *Listeria* strains depended on the initial inoculum and storage temperature. In conclusion, HPP increased the shelf-life (for 5 and 4 days, at 4 °C and 12 °C, respectively) and enhanced the safety of chicken meat.

## 1. Introduction

It is well established that poultry meat is a highly perishable food with a short shelf-life limited from 4 to 15 days under refrigeration, depending mostly on the packaging type used [1]. Microbial contamination on fresh chicken meat is diversified and mostly depends on the poultry carcass quality, the slaughtering process and the environmental microbiota [2]. Spoilage of poultry is caused only by a specific microbial association the “ephemeral/specific spoilage micro-organisms E(S)SO,” which can be introduced to the meat during processing, transportation, and storage in the market [3,4]. The metabolic activity of the specific microbiota leads to changes dependent on the availability of energy substrates i.e., low molecular weight compounds (glucose, lactate, amino acids, etc.) of meat and spoilage prevails because of accumulation of metabolic by-products [4,5]. *Brochothrix thermosphacta* can be recognized as a common spoilage microorganism often found in poultry meat [6]. *B. thermosphacta* is capable of growing at a wide range of pH (5–9), temperature (0–30 °C), or NaCl (up to 10%) and is thought to be a ubiquitous microorganism throughout the meat production chain [7]. It is responsible for spoilage of meat stored under aerobic, modified atmosphere packaging (MAP) or/and vacuum packaged conditions and can become the dominant microbiota leading to souring of the meat. During spoilage of meat, *Brochothrix* can utilize glucose and produce unpleasant off-flavors, like cheesy odors responsible for a distinct type of meat spoilage [4,8].

Apart from the E(S)SO microbiota, poultry meat might contain endogenous pathogens like *Listeria monocytogenes, Campylobacter* spp., *Staphylococcus aureus*, or *Salmonella enterica* and raw poultry products have been responsible for a significant number of cases of food poisoning to humans, especially when contaminated with *Campylobacter* spp., or *Salmonella enterica* [9,10,11]. However, since the incidence of *L. monocytogenes* in chicken meat is not required to be reported to EFSA, little information is available about the prevalence in chicken meat [11]. A recent meta-analysis study reported that *L. monocytogenes* was frequently found in chicken meat (prevalence of about 21%) at the industry or retail level and for both packed or unpacked products [11]. *L. monocytogenes* has the capacity to form biofilms and the ability to withstand acids and detergents, is resistant to chemical biocides, which result in the extended persistence of this pathogen in the industrial equipment and environment [12,13,14]. Therefore, it is of importance to perform risk assessment studies to help reveal what may be done to reduce or eliminate the pathogen prevalence in chicken meat.

High-pressure processing (HPP) is an effective nonthermal preservation technology, which is being used as an alternative food preservation method to improve food safety, to control food spoilage, and to extend the shelf-life with minimal changes in the nutritional, functional, or sensorial characteristics of the original food material [15,16,17]. Up to date, a variety of HPP products, such as ready to eat (RTE) meats, fruit juices, packaged vegetables, sea food, dairy products, sliced ham and meat are commercially available [13,15]. Depending on the product microbiota, the pressure levels that are usually used in the meat industry are between 400–600 MPa for a relatively short processing time [18,19]. Although many studies have been conducted for measuring the effect of HPP on the spoilage microbiota of red meat and meat products [20,21,22], limited information is available for raw chicken meat. In addition, the complexity of the reactions taking place in a biological system such as meat, makes it difficult to predict the effect of HPP treatments on meat microbiota, since the resistance of the different microorganisms present in meat is variable and depends on many factors (type of bacteria, food matrix, etc.,) [23]. Liu et al. [24] have investigated the pressure resistance of inoculated *Campylobacter jejuni* and *Escherichia coli* strains with or without the presence of the specific spoilage microbiota of poultry meat on aseptically prepared minced poultry meat using pressure of 400 MPa for 30 min holding time at 40 °C. The researchers reported that at this pressure level the cell counts of the inoculated (7–8 log CFU/g initial inoculum level) microorganisms (*B. thermosphacta*, *Carnobacterium divergens*, *Campylobacter jejuni*, and *Pseudomonas fluorescens*) were found below the detection limit after pressure, except the *E. coli* strain which was reduced by 3.5 log CFU/g. In spite of that, in a previous study by Argyri et al. [25] it was shown that the main spoilage microorganism after HPP (500 MPa/10 min) on chicken fillets was *B. thermosphacta*. Furthermore, many research articles have focused on the behavior of *L. monocytogenes* in meat products after HPP [26,27,28], but only a few studies have focused on chicken meat [6,29]. Therefore, it is of great importance to monitor the behavior of the pathogen after HPP on fresh chicken fillets and during shelf-life, because any cells that might survive HPP, could potentially recover and grow during storage of the product because of the psychrotrophic nature of *L. monocytogenes*.

In this context, the current study was designed to monitor the survival and growth of the indigenous *Brochothrix thermosphacta* and the inoculated *L. monocytogenes* strains after the application of HPP (500 MPa/10 min/18–20 °C) and storage of vacuum-packaged chicken fillets stored at 4 °C and 12 °C. Moreover, the shelf-life of the product was determined by sensory analysis of non-inoculated samples. Finally, the strain distribution of *Brochothrix* isolates and the inoculated *L. monocytogenes* were evaluated by using molecular tools.

## 2. Materials and Methods

### 2.1. Experimental Design and Preparation of Chicken Fillet Samples

Chicken breast fillets of two different batches were obtained from the local meat industry and transported under refrigeration to the laboratory with minimal delay, where they were held at 1 °C for 1–2 h. The meat was then aseptically cut and weighted into portions of 30 g and subsequently inoculated with *L. monocytogenes* (cocktail culture of four strains) as is described at Section 2.2. A portion of samples was not inoculated with the pathogen and served as samples for sensory analysis. All samples were packed under vacuum into plastic pouches (100 mm wide–100 mm long, 90 mm thickness), of O_2_ permeability of ca. 75 mL/m^2^/24 h/1 atm at 23 °C and 75% relative humidity of ca. 75 cc/m^2^/24 h/1 atm (Flexo–Pack S.A., Athens, Greece), using a HenkoVac 1700Machine (Howden Food Equipment B.V., The Netherlands). Half of the prepared samples were treated with HPP of 500 MPa for 10 min. All samples were stored at 4 °C and 12 °C in high precision (±0.5 °C) incubation chambers (VELP Scientifica Srl, Usmate Velate MB, Milan, Italy), for up to 14 days, until spoilage was pronounced (discoloration and presence of off–odors) and non-inoculated samples were characterized from sensory analysis as spoiled. Microbiological analysis was assessed in parallel with molecular analysis and sensory evaluation of non-inoculated samples, as is already described by Argyri et al. [25].

### 2.2. Inoculation of the Chicken Fillet Samples

Four strains of *L. monocytogenes* (FMCC–B–128, FMCC–B–129, FMCC–B–131, FMCC–B–133) were used as a cocktail to inoculate the chicken fillets at three different inoculum levels (2, 4, and 6 log CFU/g). The strains were provided by the Food Microbiology Culture Collection (FMCC), Laboratory of Microbiology and Biotechnology of Foods, Agricultural University of Athens and all strains originated from Greek food industries (FMCC–B–128 and FMCC–B–133 were isolated from soft cheese, FMCC–B–129 was isolated from RTE minced meat based frozen meal and FMCC–B–131 was isolated from conveyor belt of RTE frozen food). The strains were activated from a stock culture stored at −80 °C and were grown overnight at 37 °C in 10 mL of brain heart infusion broth (BHI, LAB049, LabM, Lancashire, UK). Each strain was harvested by centrifugation at 10,000× *g* for 5 min, washed twice with ¼ strength Ringers solution (Ringers solution tablets, 96724–100TAB, Merck, Darmstadt, Germany) and resuspended in 10 mL Ringers solution in a final concentration of approximately 9 log CFU/mL. To prepare the cocktail culture of the four strains, each strain was serially diluted on the decimal scale with ¼ strength Ringers solution and then all strains were mixed in equal volumes to give final concentrations of 2, 4 and 6 log CFU/g. Chicken fillets were then inoculated with the three inoculation levels and packed under vacuum. Half of the prepared samples were treated with HPP. To verify the counts of the inocula, the same dilutions were spread-plated on Palcam agar (Palcam agar, BK145HA, Biokar Diagnostics, Allonne, France).

### 2.3. High-Pressure Processing (HPP)

HPP experiments were conducted by applying 500 MPa for 10 min at room temperature (18–20 °C). Further details of the HPP treatment and the selection of 500 MPa/10 min, can be found at the study by Argyri et al. [25]. After the HPP treatment, the samples were divided according to their origin from the two different meat batches/different level of inoculum/HPP-treated samples/control samples (without HPP treatment) and then stored at 4 °C and 12 °C in vacuum packaging.

### 2.4. Microbiological and pH Analyses

Microbiological and pH analyses were carried out until the end of storage at 4 °C and 12 °C. From each sampling case, three packages of inoculated samples were subjected to microbiological and pH analyses and three additional packages were used for the enrichment method. Simultaneously, three samples from the non-inoculated samples were implemented in sensory evaluation. To estimate the number of viable cells, chicken fillet samples (25 g) were weighed aseptically, added to sterile ¼ Ringers solution (50 mL), and homogenized in a stomacher (Stomacher 400 Circulator, Seward Limited, Norfolk, UK) for 60 s at room temperature according to ISO 7218:2013. Serial decimal dilutions were prepared with the Ringers solution and duplicate 1 or 0.1 mL samples of the appropriate dilutions were mixed or spread on non–selective and selective media. To reduce the detection limit of the enumeration method (for spread plating) to 0.48 log CFU/g, 1 mL of the first decimal dilution was spread equally on three agar plates of each substrate. The studied agar media were the following: plate count agar (CM0325, Oxoid, Basingstoke, UK) for the enumeration of total viable counts (TVC), incubated at 30 °C for 48–72 h; de Man–Rogosa–Sharp (MRS) medium (CM 0361, Oxoid, Basingstoke, UK) adjusted to pH 5.7, overlaid with the same medium for the enumeration of LAB incubated at 30 °C for 48–72 h; streptomycin thallous acetate-actidione agar (STAA, 4020792, Biolife, Milano, Italy) with STAA selective supplement (4240052, Biolife, Milano, Italy) for the enumeration of *Brochothrix thermosphacta*, incubated at 25 °C for 72 h; rose bengal chloramphenicol agar base (LAB036, LAB M, Lancashire, UK) with selective supplement (X009, LAB M, Lancashire, UK) for the enumeration of yeasts and molds incubated at 25 °C for 48–72 h; violet red bile glucose agar (CM 0485, Oxoid, Basingstoke, UK) overlaid with the same medium, for the enumeration of *Enterobacteriaceae* incubated at 37 °C for 24 h; *Pseudomonas* agar base (CM559, Oxoid, Basingstoke, UK) supplemented with CFC selective supplement (SR0103, Oxoid, Basingstoke, UK), for the enumeration of *Pseudomonas* spp. incubated at 25 °C for 48 h; Palcam agar (BK145HA, Biokar Diagnostics, Allonne, France) supplemented with Palcam selective supplement (BS00408, Biokar Diagnostics, Allonne, France) for the enumeration of *L. monocytogenes* incubated at 37 °C for 24 and 48 h. For the HPP-treated samples, incubation time was extended by 1–2 days in all growth media to allow recovery of lethally/sublethally injured or stressed by HPP treatment cells. For the samples inoculated with *L. monocytogenes*, enrichment was performed according to ISO 11290–1:1996/Amnd1:2004 for *L. monocytogenes*. To ensure the absence of the three major pathogens at the non-inoculated samples, the samples were analyzed periodically throughout storage using the enrichment methods for *Salmonella* spp. (ISO 6579: 2002/Cor.1:2004), *E. coli* O157:H7 (ISO 16654:2001) and *L. monocytogenes* (ISO 11290–1:1996/Amd 1:2004).

The pH values were measured throughout storage with a digital pH meter (HI 2211 pH–ORP Meter, HANNA Instruments, Smithfield, RI, USA), by immersing the electrode in the meat homogenate (stomacher homogenate) after the microbiological analysis.

### 2.5. Isolation and Growth of Listeria sp. and Brochothrix sp.

Chicken meat was sampled at specific time intervals depending on the storage temperature. *Listeria* colonies were randomly isolated from plates that corresponded to the beginning (day 0) and final storage time for the two storage temperatures. From each of the aforementioned samplings, 20% of the colonies (i.e., 10–20 colonies) were randomly collected from the appropriate countable dilution on Palcam agar plates. When the pathogen was not detected using the enumeration method, random colonies (i.e., 6–8, depending on the purity) were isolated from the Palcam petri dishes from the enrichment method. *Brochothrix* colonies were randomly isolated from STAA plates that corresponded to the beginning (day 0) and to the end of the storage period for control cases of 4 °C and 12 °C, while for HPP-treated samples *Brochothrix* isolates were picked from the third day of storage and at the end of the storage period for both temperatures. Day 3 was the first day that colonies of *Brochothrix* were detected. From each of the aforementioned samplings, 20% of the colonies (i.e., 10–20 colonies) were randomly collected from the appropriate countable dilution of STAA plates. The purified cultures were stored at −80 °C in BHI broth supplemented with 20% (*v*/*v*) glycerol (Serva, Heidelberg, Germany). Before experimental use, each isolate was grown twice in BHI at 37 °C for 16 h and 25 °C for 24 h for *Listeria* and *Brochothrix* isolates respectively, while the purity of the cultures was always checked in BHI agar plates before use. The isolates (294 isolates for *Listeria* and 110 isolates for *Brochothrix*) were subsequently screened with pulsed-field gel electrophoresis (PFGE) as described in Section 2.6.

### 2.6. Pulsed-Field Gel Electrophoresis

The PFGE was applied to monitor the survival and distribution of each inoculated *L. monocytogenes* strain and also to monitor the succession of *Brochothrix* sp. isolates in the control and HPP-treated samples during storage at 4 °C and 12 °C. PFGE was performed according to Papadopoulou et al. [30]. The restriction enzyme *Apa*I (10 U) (New England Biolabs, Ipswich, MA, USA) was used for both *Listeria* and *Brochothrix* isolates, following manufacturer’s recommendation for 16 h. The restriction fragments were separated by pulsed–field gel electrophoresis on CHEF–DRII equipment (BIO–RAD, Hercules, CA, USA) in 1% (*w*/*v*) PFGE grade agarose gel (BIO–RAD, Hercules, CA, USA) in 0.5 mM tris–borate buffer and the running parameters were the following: 6 V/cm, 4 s initial switching time, 40 s final switching time and a 18 h of total run at 14 °C. Gel was stained with ethidium bromide (0.5 μg/mL) (Sigma, Missouri, USA) and bands were visualized using GelDoc system (BIO–RAD, Hercules, CA, USA). The PFGE fingerprints of *L. monocytogenes* FMCC–B128, FMCC–B129, FMCC–B131, FMCC–B133 strains were used as reference strains to compare the obtained restriction profiles of the *Listeria* isolates. For *Brochothrix* isolates, representative isolates per distinct PFGE cluster were subjected to species identification by sequencing the V1–V3 variable region of the 16S rRNA gene according to Doulgeraki et al. [31]. PCR products were purified using the QIAquick^®^ PCR Purification Kit (Qiagen, Hilden, Germany) according to the manufacturer’s instructions and sent for sequencing to CeMIA, (Department of Immunology and Histocompatibility, Faculty of Medicine, University of Thessaly, Volos, Greece). Obtained results of the 16S rRNA gene sequence were aligned with those in GenBank using the program BLAST to determine their closest known relatives to the partial 16S rRNA gene (Table S1).

### 2.7. Sensory Analysis

The sensory evaluation of the chicken fillets was performed throughout storage as previously reported [25], by a five-membered sensory panel (trained staff from the laboratory of Institute of Technology of Agricultural Products, HAO–DEMETER) at the same time intervals as for microbiological analyses. The same sensory panel was used in all evaluations and was unaware of the tested sample. The sensory assessment was conducted in triplicate under artificial light in individual booths in a special sensory analysis room allocated in the Institute of Technology of Agricultural Products and meat samples were left to reach ambient temperature prior to analysis. In brief, the sensory panel evaluated the color, smell, and taste (after cooking) of the meat in a five-point hedonic scale. Scores ranged from 1 (fresh) to 3 (unacceptable). Scores above 2 were given to characterize the meat as spoiled and indicated the end of shelf-life. No score was given by the panelists when differentiation in the sensory characteristics of the product were not linked to spoilage attributes (e.g., in the basic characteristics of the product), but to HPP treatment.

### 2.8. Statistical Analysis

All experiments were carried out in triplicate with two independent batches of chicken fillets each. Significance was established at *p* < 0.05. Results were analyzed for statistical significance with analysis of variance (ANOVA). Duncan’s multiple range test was used to determine the significant differences among results [coefficients, ANOVA tables and significance (*p* < 0.05) were computed using Statistica v.5.0 (Statsoft Inc., Tulsa, OK, USA)].

## 3. Results

### 3.1. Population Dynamics/Inactivation of the Indigenous Microbiota

**Figure 1:**
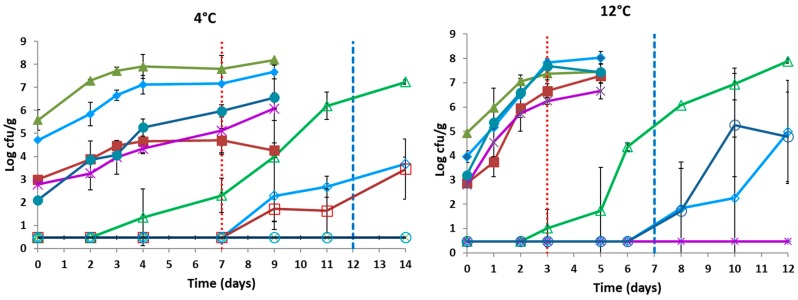
Growth curves of *B. thermosphacta* (▲) or (△), *Pseudomonas* spp. (♦) or (◊), LAB (*) or (x), *Enterobacteriaceae* (●) or (**○**)*,* yeasts/molds (■) or (□), on untreated (closed symbols), or HPP-treated (500 MPa/10 min) (open symbols) chicken fillets, respectively, during storage under vacuum package at 4 °C (left) and at 12 °C (right). Vertical red dotted line: end of the control samples shelf-life according to sensory analysis. Vertical blue dashed line: end of the HPP-treated samples shelf-life according to sensory analysis. Error bars represent the mean values ± standard deviation.

The changes of the indigenous microbiota in raw chicken fillets untreated or treated with HPP (500 MPa/10 min) during storage under vacuum package at 4 °C and 12 °C is presented in Figure 1. For the untreated samples (control), the microbiological analysis showed that the initial population of the indigenous microbiota on chicken samples stored at 4 °C and 12 °C consisted of *B. thermosphacta* (5.25 ± 0.41 log CFU/g), *Pseudomonas* spp. (4.26 ± 0.44 log CFU/g), yeasts/molds (2.92 ± 0.14 log CFU/g), LAB (2.79 ± 0.23 log CFU/g), and *Enterobacteriaceae* (2.65 ± 0.64 log CFU/g). The population of all the microbial groups on the untreated samples (without HPP) increased during storage at both temperatures, with *B. thermosphacta* (increase of 2.61 and 2.52 log CFU/g, at 4 °C and 12 °C, respectively) being the dominant microbiota followed by *Pseudomonas* spp. (increase of 2.96 and 4.07 log CFU/g, at 4 °C and 12 °C, respectively), *Enterobacteriaceae* (increase of 4.45 and 4.23 log CFU/g, at 4 °C and 12 °C, respectively), yeasts/molds (increase of 1.27 and 4.41 log CFU/g, at 4 °C and 12 °C, respectively), and LAB (increase of 3.30 and 3.86 log CFU/g at 4 °C and 12 °C, respectively) (Figure 1). Only after the end of the product’s shelf-life at 12 °C (third day of storage), *Pseudomonas* spp. and *Enterobacteriaceae* exhibited population numbers similar or higher than those of *B. thermosphacta* (Figure 1).

The population dynamics of the aforementioned microbial groups and their contribution to the final microbiota were greatly affected by the application of HPP (500 MPa/10 min), as exemplified by the evolution of all microbial counts. In brief, the application of HPP on chicken meat resulted in the reduction of all the microbial counts below the detection limit of the enumeration method, reaching a total reduction of ≥ 5 log cycles (Figure 1). During storage of the HPP-treated samples at both temperatures, the main spoilage microorganism was *B. thermosphacta*, whereas the population of the rest microbial groups was found below the detection limit of the enumeration method. However, toward and after the end of the products shelf-life a minor increase of *Pseudomonas* and yeasts/molds at 4 °C and *Pseudomonas* and *Enterobacteriaceae* at 12 °C was observed (Figure 1). *B. thermosphacta* was detected after the third day of storage at both temperatures and increased until the end of the storage period (Figure 1). Nevertheless, the final counts of *Brochothrix* population were found lower compared to those of all of the microbial groups in the control samples. Finally, the estimated shelf-life of HPP samples was 12 days at 4 °C and 7 days at 12 °C, where the counts of *B. thermosphacta* were estimated at 6 log CFU/g and 5 log CFU/g at 4 °C and 12 °C, respectively. In contrary, the end of shelf-life in control samples was after 7 days at 4 °C and 3 days at 12 °C, where *Brochothrix* population reached over 7 log CFU/g at both temperatures.

The initial pH values of chicken fillets stored at 4 °C were 5.82 ± 0.03 and 6.05 ± 0.05 for the control and the HPP–treated samples, respectively, while the corresponding values for 12 °C were 5.96 ± 0.05 and 6.14 ± 0.06, demonstrating a small increase of ca. 0.2 units (*p* < 0.05) after the application of HPP. During storage, pH values remained similar to the initial pH within the same treatment (*p* > 0.05). Nevertheless, the changes in the pH values between the different treatments were found to vary significantly during storage (*p* < 0.05), ranging between 0.1–0.23 (Figure S1).

### 3.2. Population Dynamics/Inactivation of Listeria monocytogenes

**Figure 2:**
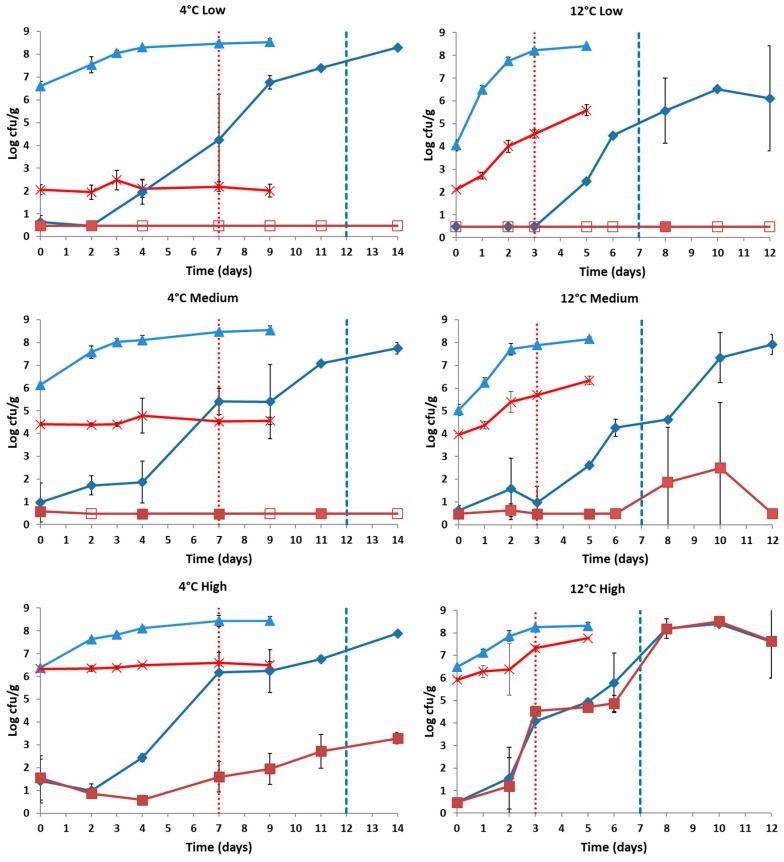
Growth curves of TVC on untreated (▲) or HPP–treated (500 MPa/10 min) (♦) chicken fillets and of *L. monocytogenes* on untreated (x) or HPP–treated (500 MPa/10 min) (■) chicken fillets inoculated with low, medium, and high inoculum level of a 4-strain cocktail of *Listeria monocytogenes* and stored under vacuum package at 4 °C (left) and at 12 °C (right). Open symbols (□) indicate absence of the pathogen after applying the enrichment method. Vertical red dotted line: end of the control samples shelf-life according to sensory analysis. Vertical blue dashed line: end of the HPP-treated samples shelf-life according to sensory analysis. Error bars represent the mean values ± standard deviation.

The survival of the pathogen *L. monocytogenes* (cocktail culture of four strains) inoculated in three different initial levels in both control and HPP-treated samples followed by storage in vacuum packaging at 4 °C and 12 °C is presented in Figure 2. Regarding the samples without HPP treatment (control), the population of the pathogen at all inocula (2, 4, and 6 log CFU/g) remained similar to the initial inoculum level in all cases during storage at 4 °C. For the samples stored at 12 °C, results showed that the population of the pathogen for the three different inocula cases showed an increase of 1.85–3.48 log CFU/g, depending on the case (Figure 2).

On the other hand, the samples that were treated with HPP and stored at 4 °C showed a decrease of the pathogen population which was depended on the different inoculum. More specifically, for the low and medium inoculum no growth of the pathogen was observed, while for the high inoculum (6 log CFU/g) a 5-log cycle reduction was recorded during cold storage. In detail, in the samples with the low initial inoculum *L. monocytogenes* was detectable after enrichment at the first days of cold storage until the fourth day that the pathogen was absent. In the samples with medium inoculum *L. monocytogenes* was found periodically present after enrichment during storage at 4 °C (Figure 2). At the high inoculum the pathogen increased during cold storage at a population level of 3 log CFU/g. At 12 °C the application of HPP resulted in the reduction of the pathogen in all the inoculation cases on day 0 to the detection limit of the enumeration method. More specifically, during storage at 12 °C pathogen population remained below the detection limit of the enumeration method for the low and medium inoculum. In the low inoculum case, *L. monocytogenes* was absent during storage at 12 °C, except from the eighth day of storage that it was detected after enrichment in one out of the six samples analyzed. Though, in the medium inoculum case *L. monocytogenes* was found always present after enrichment, while a slight increase was observed after the end of the shelf-life (seventh day) of the product. Likewise, in the high inoculum case the population of the pathogen increased reaching the level of 7 log CFU/g by the end of storage at 12 °C, as it is evident from Figure 2. Lastly, Figure 2 displays the TVC population, which represented the dominant microbiota in each case. At control samples the dominant microbiota consisted of *B. thermosphacta* and *Pseudomonas* sp., while for the HPP-treated samples TVC most likely corresponded to *B. thermosphacta*, which was found to be the main spoilage microorganism during cold storage. Similarly, at 12 °C the dominant microbiota of the control samples was *B. thermosphacta* and *Pseudomonas* sp., whereas for the HPP-treated samples TVC corresponded to *B. thermosphacta* (low and medium inoculum) and *B. thermosphacta* along with *L. monocytogenes* in the case of high inoculum.

### 3.3. Effect of HPP on the Brochothrix Isolates According to PFGE Analysis

In this study the diversity as well as the succession of *B. thermosphacta* at strain level was examined during storage after different treatments (HPP or control) and storage temperatures (4 °C and 12 °C) for chicken meat stored under vacuum packaging. *Brochothrix* isolates (52 isolates) were recovered from fresh chicken fillets (day 0) and at the end of storage for control samples at 4 °C and 12 °C, while for HPP-treated samples *Brochothrix* isolates (58 isolates) were recovered after the third day of storage and at the end of storage for both temperatures.

According to the PFGE profiles for *Brochothrix* isolates, different profiles between treatments (HPP or control) and storage temperatures (4 °C and 12 °C) were obtained. In detail, a significant diversity among isolates recovered from control samples was observed, whereas for the isolates recovered after HPP their diversity was lower. Moreover, storage conditions also affected the strain diversity resulting in a lower diversity of the isolates when samples were stored at 12 °C.

**Figure 3:**
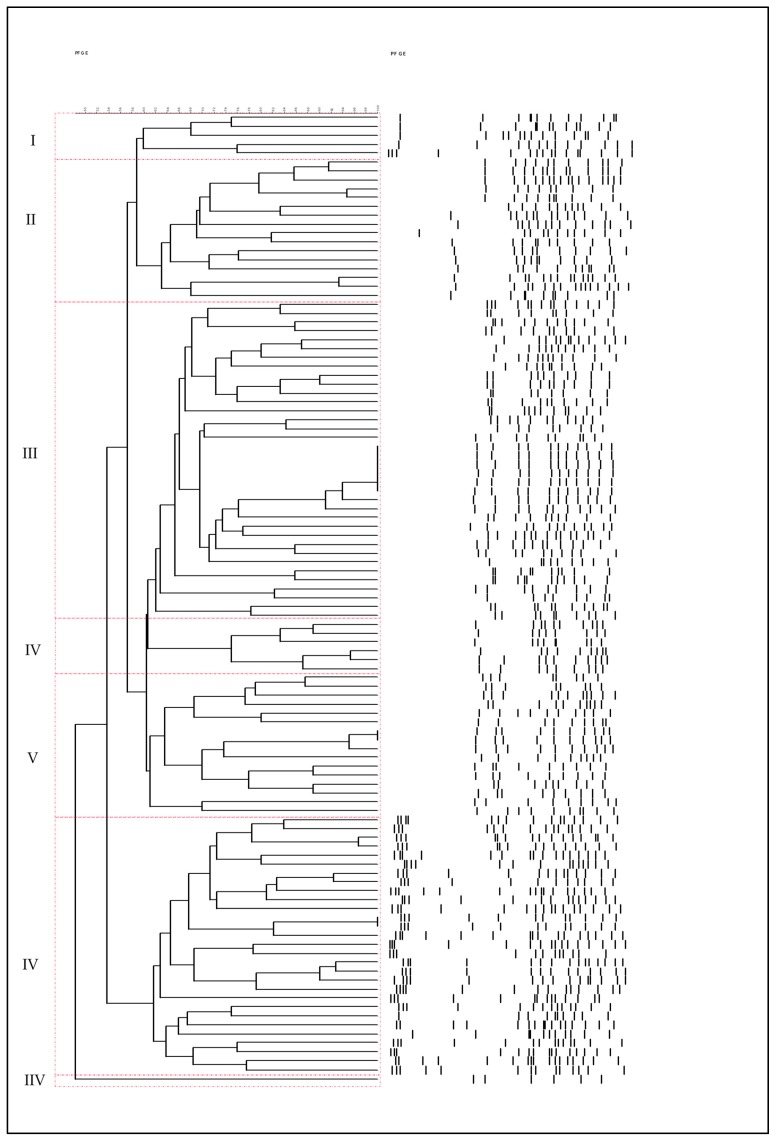
Cluster analysis of pulsed-field gel electrophoresis (PFGE) *Apa*I digestion fragments of the *Brochothrix thermosphacta* isolates calculated by the unweighted pair group method.

The dendrogram obtained after cumulative image analysis of PFGE patterns using a coefficient of similarity of 60%, resulted in seven different groups (Figure 3). In Table 1, the prevalence of the different groups related to the different conditions (untreated and treated with HPP and storage temperatures) is summarized. In detail, Group I had five isolates all recovered from the end of cold storage of HPP-treated samples. Group II encompassed 16 isolates and the diversity of the recovered PFGE profiles was observed mostly at control samples stored at both temperatures. Additionally, no isolates were recovered from control samples at 12 °C. Group III was the most abundant in terms of isolates (37). In detail, two PFGE profiles were found in HPP samples (day 3 and end of storage), while nine different profiles were recovered from the control samples at 4 °C. At 12 °C no isolates were recovered from control samples, whereas 26 isolates were recovered from HPP samples. Group IV consisted of six isolates recovered mainly from control samples for both temperatures at 0 h and at the end of storage. Only one isolate in this group was recovered from an HPP sample on the third day of storage at 4 °C. Group V contained mainly isolates (16) from control and HPP-treated samples from 12 °C. Group VI consisted of 29 isolates, where no isolates were recovered from HPP samples from 12 °C. The last group (VII) consisted of a single isolate recovered from the end of 12 °C of control samples. A representative number of isolates from the above seven groups were subjected to 16S rRNA gene sequencing, revealing that all groups were assigned to *B. thermosphacta* at a percentage over 99% (Table S1).

### 3.4. Effect of HPP on the Different Listeria Strains According to PFGE Analysis

**Figure 4:**
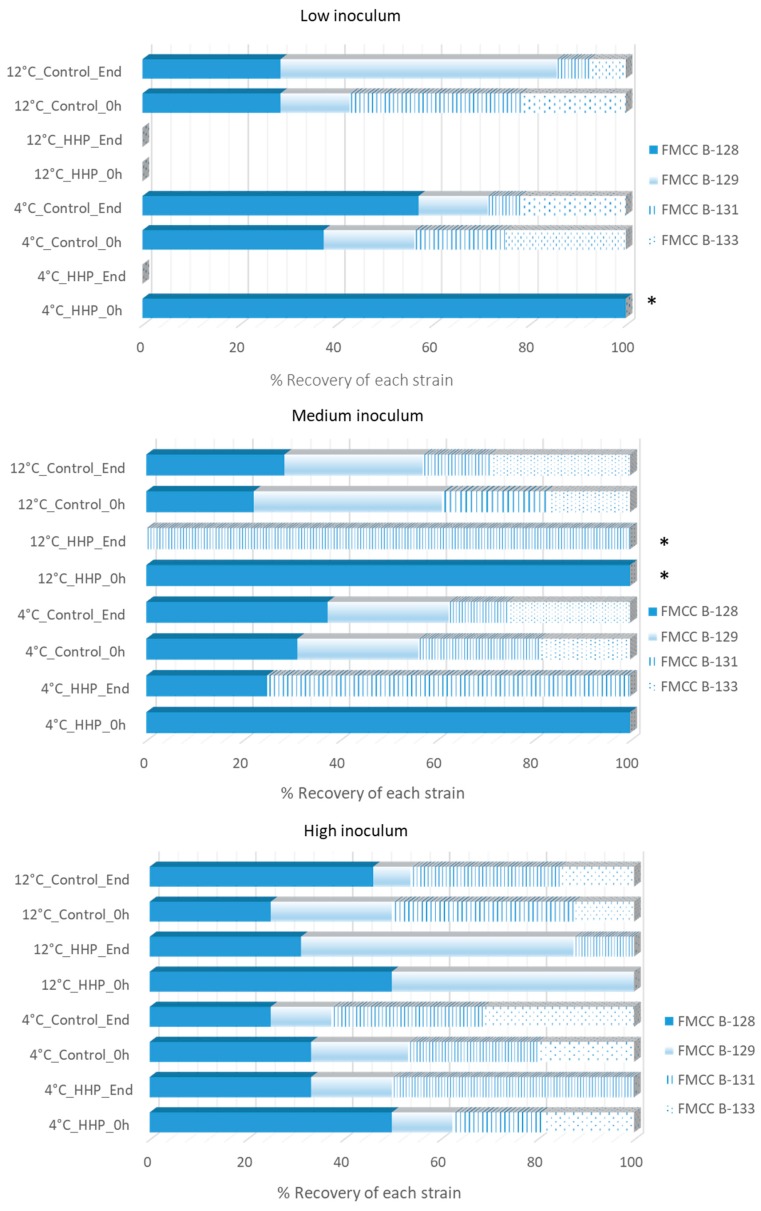
Survival of the different *L. monocytogenes* strains (FMCC–B–128, FMCC–B–129, FMCC–B–131, FMCC–B–133) on chicken fillets untreated (control) or HPP–treated (500 MPa/10 min) with low, medium, and high levels of *L. monocytogenes* initial inoculum, at the beginning (0 h) and the end of storage under vacuum package at 4 °C and 12 °C. * Pathogen detected after applying the enrichment method.

A total of 294 isolates were recovered from Palcam agar plates and the isolates were screened with PFGE to monitor the survival and the distribution of the *L. monocytogenes* strains on chicken samples untreated or treated with HPP, stored under vacuum packaging for both temperatures. The distribution of the above recovered isolates based on their PFGE profiles is presented in Figure 4. According to the PFGE analysis, the survival and distribution of the different *Listeria* strains depend on the initial inoculum level, the application of HPP along with the storage temperature (Figure 4). At 0 h for the three inoculum levels on control samples, the distribution of the strains was FMCC–B–128 (*ca.* 32–37%)> FMCC–B–131 (*ca.* 19–27%) > FMCC–B–133 (*ca.* 19–25%), and FMCC–B–129 (*ca.* 19–25%). At the end of cold storage for control samples the strain FMCC–B–128 was the dominant strain in low and medium inoculum case, while FMCC–B–131 displayed the lowest recovery percentage. Regarding the low inoculum, it has to be noted that the pathogen was detected only in samples analyzed with the enrichment method. For samples inoculated with the high inoculum strains FMCC–B–131 and FMCC–B–133 exhibited the highest recovery percentage at the end of cold storage of control samples. Subsequently, after the application of HPP the strain distribution varied between the different inoculum levels. For low (after enrichment) and medium inoculum FMCC–B–128 was the only strain recovered, whereas for the high inoculum the distribution of the strains was FMCC–B–128 (*ca.* 50%), FMCC–B–131 (*ca.* 19%), FMCC–B–133 (*ca.* 19%), and FMCC–B–129 (*ca.* 12%). At the end of storage at 4 °C of HPP-treated samples no strains were recovered from the low inoculum case (after enrichment), the strain FMCC–B–131 dominated in medium inoculum case and lastly, in the high inoculum case all strains but FMCC–B–133 were recovered.

For the samples stored at 12 °C the initial distribution of the strains for the three inocula was FMCC–B–131 (*ca.* 22–38%) > FMCC–B–129 (*ca.* 14–39%)> FMCC–B–128 (*ca.* 22–29%)> and FMCC–B–133 (*ca.* 12–22%), while at the end of storage differences were observed between the three inoculum levels in terms of recovery percentages of each strain. However, in contrast to the cold storage, no specific recovery pattern in strain distribution was observed for control samples (Figure 4). After HPP, no colonies were recovered after enrichment in the low inoculum case, FMCC–B–128 was the only recovered strain (after enrichment) in the medium inoculum case, while at high inoculum the recovered strains were FMCC–B–128 and FMCC–B–129 in a ratio of 1:1. Finally, at the end of storage at 12 °C no strains were recovered after enrichment in the low inoculum case, in the medium inoculum case the only recovered strain (after enrichment) was FMCC–B–131, while at high inoculum all strains but FMCC–B–133 were recovered. The latter finding is of interest, since the same strains were absent or present in the high inoculum case after cold storage of HPP–treated samples. For comparison reasons, it has to be noted that in the cases that the isolates were recovered after enrichment the presence of each strain has to be characterized as random and the appearance of the strains in the figures should be interpreted as qualitative information rather than % contribution in the samples [25].

### 3.5. Sensory Analysis

The organoleptic changes of the control and HPP-treated (non–inoculated) samples were similar with our previous published work for chicken fillets stored at 4 °C and 12 °C under vacuum packaging [25]. In brief, the time required by the sensory panel to consider a sample as spoiled, differed for each temperature and for HPP-treated or untreated (control) samples. Concerning cold storage, the shelf-life of the HPP-treated chicken fillets was increased to 12 days of storage, while for control samples the end of shelf-life was estimated after 7 days of storage (Figure 1). In addition, at the time of the sensory rejection the TVC values were ca. 7.5 log CFU/g and 6.0 log CFU/g for the control and HPP-treated samples, respectively (Figure 1). At 12 °C meat was characterized as spoiled by the sensory panel at the third day of storage for control samples and at the seventh day of storage for HPP-treated samples (Figure 2). The mean values of TVC at the time of sensory rejection (meat characterized as spoiled) were ca. 8.0 log CFU/g and 5.2 log CFU/g for control and HPP-treated samples, respectively (Figure 2). The organoleptic attribute that was mostly affected by the HPP treatment was the color of the product. In more details, panelists mentioned that in the raw chicken the color was found to be whiter with increased lightness, but after cooking the appearance of the product was similar to the control. However, the juiciness and coherence were improved in the case of HPP-treated samples, characteristics that were pointed out by all the panelists.

## 4. Discussion

The microbiological results obtained in this study demonstrated similar or higher reductions in the population of indigenous microbiota after the application of HPP, as compared to literature findings of poultry products or other meat products treated with HPP. In more details, in the current work *B. thermosphacta* was found to be the dominant microorganism after HPP, a result that was also observed by Argyri et al. [25], while a small increase of *Pseudomonas* spp. and yeasts/molds at 4 °C and *Pseudomonas* and *Enterobacteriaceae* at 12 °C was observed after the end of the shelf-life of the HPP-treated chicken fillets. Previous studies have reported that HPP caused a reduction in the population of the aerobic and anaerobic mesophiles, LAB, *Listeria* spp., *Staphylococcus* spp., *B. thermosphacta*, coliforms, yeasts, and molds to below the detection limit of the enumeration method on a variety of RTE meat products at 600 MPa/180 sec [20], and to levels below the detection limit of enumeration method for *B. thermosphacta*, *Carnobacterium divergens*, *Campylobacter jejuni* and *Ps. fluorescens* after 400 MPa/30 min on sterile minced poultry meat [24]. A reduction of 3.7 log CFU/g in the aerobic mesophiles of the mechanically recovered poultry meat at 450 MPa/15 min was observed by Yuste et al. [32]. Also, in the recent work by Al–Nehlawi et al. [6] poultry sausages were inoculated with 7–9 log CFU/g of various monocultures of the common meat microbiota, HPP was applied (350 MPa/10 min) and then products were packed without or with CO_2_ and stored at 2 °C. From the results it was evident that the application of HPP has resulted in a significant reduction of 4 log CFU/g of *Leuconostoc carnosum*, while in samples inoculated with *B. thermosphacta* the population of this bacterium suffered a reduction of 5 log CFU/g and no recovery was observed throughout the tested poultry products packed with CO_2_. Additionally, in another study *B. thermosphacta* was inactivated after HPP (400 MPa/10 min) when initial population of the samples was ca. 4.5 log CFU/g on a sliced packed cooked ham stored in vacuum packages under refrigeration temperatures [33]. Finally, Rodríguez–Calleja et al. [34] studied the multiple effect of HPP, MAP, and edible coatings on the microbiota of chicken fillets stored at 4 °C and the results demonstrated that *B. thermosphacta* was inactivated when all hurdles were applied, although this bacterium consisted of 89% of the primary microbiota. When less hurdles were applied (MAP and HPP treatment) *B. thermosphacta* was capable of growing and becoming the dominant microbiota throughout storage [34]. In the current study, HPP increased the shelf-life of chicken fillets for 5 and 4 days, at 4 °C and 12 °C, respectively, which was similar to Argyri et al. [25] previous findings for cold storage (6 days) but different for 12 °C (2 days). It has to be noted that besides the fact that the trained sensory panel consisted of a limited number of persons, they all agreed for the end of the product’s shelf-life (sensory scores ≥ 2).

As mentioned above, *B. thermosphacta* was found to be the dominant population after the application of HPP on chicken meat. Accordingly, PFGE was used to study the diversity, as well as, the succession of *B. thermosphacta* isolates under different treatments (untreated and treated with HPP) and temperatures (4 °C and 12 °C) during storage of chicken meat. Results showed that *Brochothrix* isolates exhibited a significant diversity among control samples, whereas this was limited for the HPP-treated samples. These results were in line with previous studies in meat and meat products [33,35], where HPP treatment was found to decrease the strain diversity. Moreover, storage at abuse temperatures (12 °C) affected the strain diversity resulting in a lower diversity. Similar results were also observed in other studies dealing with meat spoilage at various temperatures [30,31]. *B. thermosphacta* has gained much attention because of its ubiquitous nature and its potential to dominate as a food spoiler on meat and seafood products stored under MAP. For instance, *B. thermosphacta* was found to be one of the dominant species in French chicken cuts stored under various MAP concentrations after a 16S rRNA gene pyrosequencing approach in the study of Rouger et al. [1]. At the aforenoted study, the importance of the slaughterhouse environment in the diversity of the microbiota was also discussed. This result was also evident in the work by Samapundo et al. [36], where it was shown that *B. thermosphacta* was isolated from cutting blades and leg hooks used for the preparation of chicken cuts. Recent studies dealt with the typing of *Brochothrix* sp. isolates in an effort to evaluate genotypic and phenotypic diversity of this species and finally correlate spoilage potential of the isolates [37,38]. Illikoud et al. [37] evaluated different molecular methods for the typing of 161 *Brochothrix* isolates from different foods and results showed that *Brochothrix* isolates that were derived from different ecological and geographical origins were widely distributed in all the groups. In addition, the formed groups encompassed isolates from different animals, or/and for fresh and spoiled food, or/and from raw and processed food and suggested that the strains did not share any ecotype. Finally, from both studies it was shown that the spoilage potential of *Brochothrix* isolates is mostly strain dependent [37,38].

In the current study, a cocktail culture of four strains of *Listeria monocytogenes* in three inoculum levels was used to inoculate chicken fillets to acquire information regarding microbial risk assessment. After the application of HPP the pathogen population decreased depending on the inoculum case. For the low and medium inoculum case the pathogen was detected throughout the shelf-life at both temperatures in populations near to the detection limit or after enrichment. However, the pathogen decreased ≥ 5-log cycles after HPP and subsequently increased to 1.6 and 4.5 log CFU/g at 4 °C and 12 °C, respectively, by the end of shelf-life in the high inoculum case. Many studies have confirmed that HPP treatments sub-lethally injure microorganisms, but they are capable of partial recovery and growth after some days of storage, as it was evident in this study too. So far, several studies have focused on the effect of HPP on *L. monocytogenes* in a variety of meats and meat products. In a study by Jofre et al. [39] cooked ham was inoculated with three strains of *L. monocytogenes* (4 log CFU/g initial inoculum level) among other pathogens and pressurized at 600 MPa/5 min at 10 °C. From the results it was evident that the pathogen population was reduced more than 3.5 log CFU/g and remained at levels below 10 CFU/g during refrigerated storage [39]. Similar results were observed in the study of Hayman et al. [20] in a variety of RTE meats inoculated with 4 log CFU/g of *L. monocytogenes*, where after HPP treatment (600 MPa/180 s/20 °C) *L. monocytogenes* was found sporadically present after enrichment for a storage period of 91 days at 4 °C. Reduction of 7 log units of a 3-strain cocktail of *L. monocytogenes* (7 log CFU/g initial inoculum level) was observed at 600 MPa/3 min on cooked ham in the study of Bover-Cid et al. [40]. Yet, the cells that survived were able to initiate growth just after HPP treatment [40]. Schneinberg et al. [41] reported that inoculated beef jerky which was treated with two cycles of HPP (550 MPa/60 sec/22 °C) resulted in a reduction of approximately 1 log of *L. monocytogenes* (7 log CFU/g initial inoculum level) and subsequently it increased during storage under vacuum packaging, in contrast to the other examined inoculated pathogens that exhibited a significant reduction in the population levels. Marcos et al. [42] studied the effect of HPP in tandem with antimicrobial packaging films on cooked ham and reported that HPP (400 MPa/10 min) reduced the initial level of *L. monocytogenes* population from 4 log CFU/g to 0.6 log CFU/g and the latter population remained stable throughout storage at 6 °C. However, in the aforementioned study the application of HPP without the use of antimicrobial packaging was found inefficient to prevent pathogen growth during storage at 6 °C [42]. In other studies, pressures less than 400 MPa and holding times of more than 10 min did not result in a meaningful reduction of *L. monocytogenes* in a variety of meat products [43,44]. Finally, Kruk et al. [45] inoculated sterilized chicken breast fillets with 7 log CFU/g of *L. monocytogenes*, *S*. Typhimurium, and *E. coli* and applied three different pressure treatments (300, 450, and 600 MPa/5 min/15 °C). More than a 7 log cycle reduction was achieved at 450 and 600 MPa, while at 300 MPa cell counts were reduced by 4 log CFU/g. *L. monocytogenes* was not detected in samples pressurized with 450 and 600 MPa during storage at 4 °C, but its population remained close to 4 log CFU/g in samples treated with 300 MPa [45]. According to the above, it was evident that inactivation of *L. monocytogenes* varies and depends on the product type, the different HPP treatments, and most likely the different strains tested.

Several studies have monitored the survival of *L. monocytogenes* strains, however, to our knowledge, no work until now has implemented in typing with molecular methods the survival of *Listeria* strains after HPP treatment, although, the survival of different strains of the pathogen has been studied when pathogen was inoculated either as a monoculture [46,47] or as mixed culture [40] in a variety of products after different HPP treatments. Thus, PFGE typing revealed the distribution of the inoculated pathogenic strains and it was evident that pathogen survival was strain dependent. Each strain behaved different in relation to the different inoculum level, storage temperature, and HPP treatment (untreated or HPP–treated samples). For instance, the strain FMCC–B–128 was found to be the main recovered strain after HPP, however, at the end of storage for both temperatures, the aforementioned strain exhibited the lowest recovery percentages, while the control showed no specific recovery pattern for the aforenoted strain. Moreover, in the high inoculum case the same strains were absent or present at both storage temperatures in HPP–treated samples. According to literature findings, in a variety of products inoculated with different strains of *L. monocytogenes* the survival of *L. monocytogenes* was found to be strain-dependent and was affected by a variety of temperatures used for storage [48,49,50].

## 5. Conclusions

The aim of this study was to evaluate the synergistic effect of combining vacuum packaging and HPP treatment in reducing or inactivating spoilage and pathogenic bacteria (*Listeria monocytogenes*), as well as, monitoring the shelf-life of chicken fillets. Results demonstrated that HPP increased the shelf-life and enhanced the safety of chicken meat. Additionally, the microbiota of HPP-treated samples consisted only of a single species, i.e., *B. thermosphacta*. Strain diversity of *Brochothrix* isolates was affected by HPP treatment leading to a lower diversity of the pressed samples, while strain diversity of the pathogen was mostly depended on the inoculum level and storage temperature conditions. However, further research is needed to better understand the inhibitory effects of HPP on target strains of chicken microbiota.

## Figures and Tables

**Table 1:** Distribution of *B. thermosphacta* isolates at the beginning and the end of storage under vacuum package of chicken fillets at 4 °C and 12 °C untreated (control) or treated with HPP (500 MPa/10 min).

T (°C)	Treatment	Storage Time	Group							
			I	II	III	IV	V	VI	VII	Total Isolates
4 °C	Control	Beginning	0	2 ^a^	2	1	3	3	0	11
		End	0	4	7	1	0	3	0	15
	HPP	Beginning ^b^	0	4	0	1	0	7	0	12
		End	5	1	2	0	0	6	0	14
12 °C	Control	Beginning	0	0	0	2	6	6	0	14
		End	0	0	0	1	6	4	1	12
	HPP	Beginning ^b^	0	2	7	0	1	0	0	10
		End	0	3	19	0	0	0	0	22
Total isolates			5	16	37	6	16	29	1	110

^a^: number of isolates. ^b^: colonies isolated on day 3 (first appearance of colonies on STAA– no colonies were grown at day 0).

## Supplementary Materials

The following are available online at https://www.mdpi.com/2076-2607/7/11/520/s1.

## References

[1] Rouger A., Moriceau N., Prevost H., Remenant B., Zagorec M. (2018). Diversity of bacterial communities in French chicken cuts stored under modified atmosphere packaging. Food Microbiol..

[2] Chaillou S., Chaulot-Talmon A., Caekebeke H., Cardinal M., Christieans S., Denis C., Desmonts M.H., Dousset X., Feurer C., Hamon E. (2015). Origin and ecological selection of core and food-specific bacterial communities associated with meat and seafood spoilage. ISME J..

[3] Argyri A.A., Panagou E.Z., Nychas G.J.E., Kerry J.P. (2012). Advances in traditional, vacuum and modified atmosphere packaging MAP of fresh and processed poultry products. Advances in Meat, Poultry and Seafood Packaging.

[4] Nychas G.J.E., Skandamis P.N., Tassou C.C., Koutsoumanis K.P. (2008). Meat spoilage during distribution. Meat Sci..

[5] Nychas G.J.E., Skandamis P.N. (2005). Fresh meat spoilage and modified atmosphere packaging MAP. Improving the Safety of Fresh Meat. Woodhead Publ..

[6] Al-Nehlawi A., Guri S., Guamis B., Saldo J. (2014). Synergistic effect of carbon dioxide atmospheres and high hydrostatic pressure to reduce spoilage bacteria on poultry sausages. LWT Food Sci. Technol..

[7] Stanborough T., Fegan N., Powell S.M., Tamplin M., Chandry P.S. (2017). Insight into the.genome of *Brochothrix thermosphacta*, a problematic meat spoilage bacterium. Appl. Environ. Microbiol..

[8] Pennacchia C., Villani F., Ercolini D. (2009). Development of a Real-Time PCR assay for the specific detection of *Brochothrix thermosphacta* in fresh and spoiled meat. Int. J. Food Microbiol..

[9] Del Olmo A., Calzada J., Nunez M. (2012). Effect of lactoferrin and its derivatives, high hydrostatic pressure, and their combinations, on *Escherichia coli* O157:H7 and *Pseudomonas fluorescens* in chicken filets Innovative. Innov. Food Sci. Emerg. Technol..

[10] EFSA European Food Safety Authority. https://www.efsa.europa.eu/en/efsajournal/pub/5077.

[11] Gonçalves-Tenório A., Nunes Silva B., Rodrigues V., Cadavez V., Gonzales-Barron U. (2018). Prevalence of Pathogens in Poultry Meat: A Meta-Analysis of European Published Surveys. Foods.

[12] Allen K.J., Walecka-Zacharska E., Chen J.C., Kosek-Paszkowska K., Devlieghere F., Van Meervenne E., Osek J., Wieczorek K., Bania J. (2018). *Listeria Monocytogenes*: An Examination of Food Chain Factors Potentially Contributing to Antimicrobial Resistance. Food Microbiol..

[13] Ferreira M., Almeida A., Delgadillo I., Saraiva J., Cunha A. (2016). Susceptibility of *Listeria monocytogenes* to high pressure processing: A review. Food Rev. Int..

[14] Zhang Q.Q., Han Y.Q., Cao J.X., Xu X.L., Zhou G.H., Zhang W.Y. (2012). The spoilage of air-packaged broiler meat during storage at normal and fluctuating storage temperatures. Poultry Sci..

[15] Huang H.W., Wu S.Z., Lu J.K., Shyu Y.T., Wang C.Y. (2017). Current status and future trends of high-pressure processing in food industry. Food Control.

[16] Bravo D., de Alba M., Medina M. (2014). Combined treatments of high-pressure with the lactoperoxidase system or lactoferrin on the inactivation of *Listeria monocytogenes*, *Salmonella* Enteritidis and *Escherichia coli* O157:H7 in beef carpaccio. Food Microbiol..

[17] Hugas M., Garriga M., Monfort J.M. (2002). New mild technologies in meat processing: High pressure as a model technology. Meat Sci..

[18] Chien S.Y., Sheen S., Sommers C.H., Sheen L.Y. (2016). Modeling the Inactivation of Intestinal Pathogenic Escherichia coli O157:H7 and Uropathogenic E. coli in Ground Chicken by High Pressure Processing and Thymol. Front. Microbiol..

[19] Simonin H., Duranton F., de Lamballerie M. (2012). New Insights into the High-Pressure Processing of Meat and Meat Products. Comp. Rev. Food Sci. Food Saf..

[20] Hayman M.M., Baxter I., O’Riordan P.J., Stewart C.M. (2004). Effects of High-Pressure Processing on the Safety, Quality, and Shelf Life of Ready-to-Eat Meats. J. Food Prot..

[21] Jofré A., Aymerich T., Grebol N., Garriga M. (2009). Efficiency of high hydrostatic pressure at 600 MPa against food-borne microorganisms by challenge tests on convenience meat products. LWT Food Sci. Technol..

[22] Lerasle M., Guillou S., Simonin H., Anthoine V., Chéret R., Federighi M., Membré J.-M. (2014). Assessment of *Salmonella* and *Listeria monocytogenes* level in ready-to-cook poultry meat: Effect of various high pressure treatments and potassium lactate concentrations. Int. J. Food Microbiol..

[23] Rendueles E., Omer M.K., Alvseike O., Alonso-Calleja C., Capita R., Prieto M. (2011). Microbiological food safety assessment of high hydrostatic pressure processing: A review. LWT Food Sci. Technol..

[24] Liu Y., Betti M., Ganzle M.G. (2012). High Pressure Inactivation of *Escherichia coli, Campylobacter jejuni*, and Spoilage Microbiota on Poultry Meat. J. Food Prot..

[25] Argyri A.A., Papadopoulou O.S., Nisiotou A., Tassou C.C., Chorianopoulos N. (2018). Effect of high pressure processing on the survival of *Salmonella* Enteritidis and shelf-life of chicken fillets. Food Microbiol..

[26] Hereu A., Dalgaard P., Garriga M., Aymerich T., Bover-Cid S. (2014). Analyzing and modelling the growth behavior of *Listeria monocytogenes* on RTE cooked meat products after a high pressure treatment at 400 MPa. Int. J. Food Microbiol..

[27] Rubio B., Possas A., Rincon F., García-Gímeno R.M., Martínez B. (2018). Model for *Listeria monocytogenes* inactivation by high hydrostatic pressure processing in Spanish chorizo sausage. Food Microbiol..

[28] Valdramidis V.P., Patterson M.F., Linton M. (2015). Modelling the recovery of *Listeria monocytogenes* in high pressure processed simulated cured meat. Food Control.

[29] Stratakos A.C.H., Linton M., Patterson M.F., Koidis A. (2015). Effect of high-pressure processing on the shelf life, safety and organoleptic characteristics of lasagne ready meals during storage at refrigeration and abuse temperature. Innov. Food Sci. Emerg. Technol..

[30] Papadopoulou O.S., Doulgeraki A.I., Botta C., Cocolin L., Nychas G.J.-E. (2012). Genotypic characterization of *Brochothrix thermosphacta* isolated during storage of minced pork under aerobic or modified atmosphere packaging conditions. Meat Sci..

[31] Doulgeraki A.I., Paramithiotis S., Nychas G.-J.E. (2011). Characterization of the *Enterobacteriaceae* community that developed during storage of minced beef under aerobic or modified atmosphere packaging conditions. Int. J. Food Microbiol..

[32] Yuste J., Capellas M., Fung D.Y.C., Mor-Mur M. (2001). Inactivation and sublethal injury of foodborne pathogens by high pressure processing: Evaluation with conventional media and thin agar layer method. Food Res. Int..

[33] Han Y., Jiang Y., Xu Y., Sun X., Xu B., Zhou G. (2011). Effect of high pressure treatment on microbial populations of sliced vacuum-packed cooked ham. Meat Sci..

[34] Rodríguez-Calleja M., Cruz-Romero M.C., O’Sullivan M.G., García-López M.L., Kerry J.P. (2012). High-pressure-based hurdle strategy to extend the shelf-life of fresh chicken breast fillets. Food Control.

[35] Han Y., Xu Y., Jiang Y., Zhou G., Sun X., Xu B. (2010). Inactivation of food spoilage bacteria by high pressure processing: Evaluation with conventional media and PCR–DGGE analysis. Food Res. Int..

[36] Samapundo S., de Baenst I., Aerts M., Cnockaert M., Devlieghere F., Van Damme P. (2019). Tracking the sources of psychrotrophic bacteria contaminating chicken cuts during processing. Food Microbiol..

[37] Illikoud N., Rossero A., Chauvet R., Courcoux P., Pilet M.-F., Charrier T., Jaffres E., Zagorec M. (2019). Genotypic and phenotypic characterization of the food spoilage bacterium *Brochothrix thermosphacta*. Food Microbiol..

[38] Casaburi A., De Filippis F., Villani F., Ercolini D. (2014). Activities of strains of *Brochothrix thermosphacta* in vitro and in meat. Food Res. Int..

[39] Jofré A., Garriga M., Aymerich T. (2008). Inhibition of *Salmonella* sp. *Listeria monocytogenes* and *Staphylococcus aureus* in cooked ham by combining antimicrobials, high hydrostatic pressure and refrigeration. Meat Sci..

[40] Bover-Cid S., Serra-Castellóa C., Dalgaard P., Garriga M., Jofré A. (2019). New insights on *Listeria monocytogenes* growth in pressurised cooked ham: A piezo-stimulation effect enhanced by organic acids during storage. Int. J. Food Microbiol..

[41] Scheinberg J.A., Svoboda A.L., Cutter C.N. (2014). High-pressure processing and boiling water treatments for reducing *Listeria monocytogenes*, *Escherichia coli* O157:H7, *Salmonella* spp.; and *Staphylococcus aureus* during beef jerky processing. Food Control.

[42] Marcos B., Jofre A., Aymerich T., Monfort J.P., Garriga M. (2008). Combined effect of natural antimicrobials and high pressure processing to prevent *Listeria monocytogenes* growth after a cold chain break during storage of cooked ham. Food Control.

[43] Balamurugan S., Ahmed R., Chibeu A., Gao A., Koutchma T., Strange P. (2016). Effect of salt types and concentrations on the high-pressure inactivation of *Listeria monocytogenes* in ground chicken. Int. J. Food Microbiol..

[44] Bover-Cid S., Belletti N., Garriga M., Aymerich T. (2011). Model for *Listeria monocytogenes* inactivation on dry-cured ham by high hydrostatic pressure processing. Food Microbiol..

[45] Kruk Z.A., Yun H., Rutley D.L., Lee E.J., Kim Y.J., Jo C. (2011). The effect of high pressure on microbial population, meat quality and sensory characteristics of chicken breast fillet. Food Control.

[46] Evert-Arriagada K., Trujilloa A.G., Amador-Espejob G.G., Hernández-Herreroa M.M. (2018). High pressure processing effect on different *Listeria* spp. in a commercial starter-free fresh cheese. Food Microbiol..

[47] Jofré A., Aymerich T., Bover-Cid S., Garriga M. (2010). Inactivation and recovery of *Listeria monocytogenes*, *Salmonella enterica* and *Staphylococcus aureus* after high hydrostatic pressure treatments up to 900 MPa. Int. Microbiol..

[48] Kagli D.M., Iliopoulos V., Stergiou V., Lazaridou A., Nychas G.J. (2009). Differential *Listeria monocytogenes* Strain Survival and Growth in Katiki, a Traditional Greek Soft Cheese, at Different Storage Temperatures. Appl. Environ. Microbiol..

[49] Papadopoulou O., Chorianopoulos N. (2016). Production of a functional fresh cheese enriched with the probiotic strain *Lb. plantarum* T571 isolated from traditional Greek product. Curr. Res. Nutr. Food Sci..

[50] Papadopoulou O.S., Argyri A.A., Varzakis E.E., Tassou C.C., Chorianopoulos N.G. (2018). Greek functional Feta cheese: Enhancing quality and safety using a *Lactobacillus plantarum* strain with probiotic potential. Food Microbiol..

