# Supplementary material for: Quality and Safety of Fresh Chicken Fillets after High Pressure Processing: Survival of Indigenous Brochothrix thermosphacta and Inoculated Listeria monocytogenes
Source: Microorganisms. 2019 Nov 2;7(11):520. doi: 10.3390/microorganisms7110520 (PMC6921100; doi:10.3390/microorganisms7110520)
Supplement: Supplementary file 1 [file microorganisms-07-00520-s001.pdf]

Table S1: Identity of *Brochothrix* isolates after sequencing the V1-V3 variable region of 16S rRNA gene.

Isolate	Closest Relative	Identity	Accession no. of closest relative
BC9	*Brochothrix thermosphacta*	99%	AY543017
BC28	*Brochothrix thermosphacta*	99%	AY543017
BP1	*Brochothrix thermosphacta*	99%	AY543017
BP2	*Brochothrix thermosphacta*	100%	CP023483
BP12	*Brochothrix thermosphacta*	99%	CP023483
BTC2	*Brochothrix thermosphacta*	100%	AY543017
BTC12	*Brochothrix thermosphacta*	100%	CP023643
BTC47	*Brochothrix thermosphacta*	100%	AY543022
BTP14	*Brochothrix thermosphacta*	99%	CP023483
BTP18	*Brochothrix thermosphacta*	99%	AY543017

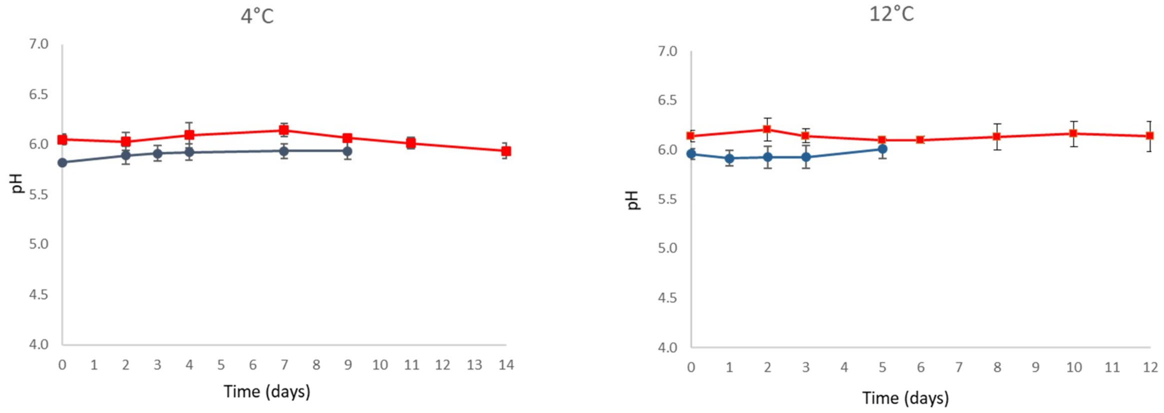

Figure S1: Changes in the pH values during storage of chicken fillets untreated (●) or HPP–treated (500MPa/10min) (■) during storage under vacuum packaging at 4˚C (right) and at 12˚C (left). Error bars represent mean values ± standard deviation.
